# Oral Manifestations and Maxillo-Facial Features in the Acromegalic Patient: A Literature Review

**DOI:** 10.3390/jcm11041092

**Published:** 2022-02-18

**Authors:** Alberto De Stefani, Francesca Dassie, Alexandra Wennberg, Giorgia Preo, Alice Muneratto, Roberto Fabris, Pietro Maffei, Antonio Gracco, Giovanni Bruno

**Affiliations:** 1 Faculty of Dentistry, University of Padova, 35128 Padova, Italy; alberto.de.stefani@hotmail.it (A.D.S.); giorgiapreo@gmail.com (G.P.); alicemuneratto1996@gmail.com (A.M.); antonio.gracco@unipd.it (A.G.); 2 Department of Medicine, University of Padova, 35128 Padova, Italy; francesca.dassie@unipd.it (F.D.); roberto.fabris@aopd.veneto.it (R.F.); pietro.maffei@unipd.it (P.M.); 3 Unit of Epidemiology, Institute of Environmental Medicine, Karolinska Institutet, 17177 Stockholm, Sweden; amvwennberg.unipd@gmail.com

**Keywords:** acromegaly, oral manifestations acromegaly, dental manifestations acromegaly, acromegalic features, oro-dental acromegaly, dentist acromegaly, acromegaly oro-dental diagnosis

## Abstract

**Background:** Acromegaly is a chronic disease caused by an abnormal secretion of growth hormone (GH) by a pituitary adenoma, resulting in an increased circulating concentration of insulin-like growth factor 1 (IGF-1). The main characteristics are a slow progression of signs and symptoms, with multisystemic involvement, leading to acral overgrowth, progressive somatic changes, and a complex range of comorbidities. Most of these comorbidities can be controlled with treatment. The literature reveals that the most evident and early signs are those related to soft tissue thickening and skeletal growth, especially in the head and neck region. **Methods:** The authors reviewed the available literature on the clinical oro-dental features of acromegaly, selecting articles from PubMed and Google Scholar. The aim of this review was to summarize all the reported clinical oro-dental features of acromegalic patients. **Results:** The most common facial dimorphisms involved the maxillo-facial district, with hypertrophy of the paranasal sinuses, thickening of the frontal bones, and protruding glabella, which may be associated with joint pain and clicks. Regarding the oro-dental signs, the most frequent are dental diastema (40–43%), mandibular overgrowth (22–24%), mandibular prognathism (20–22%), and macroglossia (54–58%). These signs of acromegaly can be significantly reduced with adequate treatment, which is more effective when initiated early. **Conclusions:** Increased awareness of acromegaly among dentists and maxillo-facial surgeons, along with the early identification of oro-facial changes, could lead to an earlier diagnosis and treatment, thereby improving patients’ quality of life and prognosis.

## 1. Introduction

Acromegaly is a rare chronic disease caused by an excessive secretion of growth hormone (GH), mostly due to an adenoma of the anterior pituitary gland and resulting in an increased circulating concentration of insulin-like growth factor 1 (IGF-1), the main effector of GH activity. It is characterized by a slow progression of signs and symptoms and multisystemic involvement, leading to physical alterations, progressive somatic changes, and a complex range of systemic comorbidities [1,2,3,4,5,6,7,8,9,10].

The somatic feature changes are slow and often cause a delay in diagnosis; facial dysmorphism, oro-dental signs, enlargement of extremities, and soft tissue thickening are the most common and earliest manifestations of acromegaly [11,12,13,14,15,16]. In the literature, soft tissue thickening and skeletal growth, especially in the head and neck region, are reported as the most evident signs and symptoms of the disease [17,18,19,20,21,22,23,24]. The prevalence and evolution of these signs and symptoms during the different disease phases (i.e., active disease, controlled diseased, and cured) is widely debated. [19]. However, oral changes can develop early and are often reported as the first symptoms of the disease, present at diagnosis in many patients [22]. 

Focusing on facial changes and oro-dental alterations, these manifestations, in particular, the development of malocclusion due to mandibular prognathism, are associated with reduced self-esteem and decreased quality of life (QoL) [19,25], and so often lead patients to seek treatment. Consequently, especially if the specialists are adequately trained, these signs can lead to an early diagnosis of acromegaly [19]. However, despite the high prevalence of oral and facial dimorphisms and the early onset of these manifestations, specialists such as dentists and orthodontists have not yet played a substantial role in diagnosis. If dentists and orthodontists could reliably recognize these early signs of acromegaly, it would likely lead to earlier diagnosis of the disease in many patients. The subsequent earlier initiation of treatment for these patients could lead to a substantially improved prognosis and QoL. Here we aim to describe oral and facial changes in acromegaly patients and increase awareness among care providers, including dentists, dental hygienists, and orthodontists.

## 2. Methods

The authors reviewed the available literature and selected articles using PubMed, Web of Science and Google Scholar. The following keywords were entered separately and in combination: “acromegaly,” “oral manifestations,” “dental manifestations,” “dental manifestation,” “oro-dental acromegaly,” “facial manifestation of acromegaly,” “dentist,” “dentist acromegaly,” and “orthodontics.”

All articles published between 1950 and 2021 were considered for inclusion. We included only papers published in English. We excluded case reports and papers for which only the abstract was available. The literature was analyzed specifically for the collection and rationalization of all the reported clinical oro-dental features shown by the acromegalic patients. The aim of this review was to collect all the clinical oro-dental features observed and reported in acromegalic patients.

## 3. Acromegaly

Acromegaly prevalence ranges from 20 to 130 people per million inhabitants [2,4,7,8,9,10,11,12,13,14,15], with an annual incidence of 2 to 11 cases per million people [4,7,8,9,10,13,15]. Most studies show that there are no sex differences in acromegaly prevalence [2,4,6,16]; however, some studies have shown a slightly higher prevalence among women [12]. The mean age at the time of diagnosis ranges from 40 to 50 years [2,6,8,9,12,16], with younger patients tending to have a more aggressive disease [16]. GH excess in children and gigantism are mostly due to genetic causes, such as multiple endocrine neoplasia, Carney complex, and familial isolated pituitary adenoma [3]. The mortality associated with acromegaly has decreased over time, from a standardized mortality rate of 1.76 in 2008 to 1.35 since 2010 [3].

The most frequent signs and symptoms of acromegaly are: menses abnormalities, physical changes (e.g., acral overgrowth), headache, paresthesia, impaired glucose metabolism, erectile dysfunction, arthropathy, hypertension, fatigue, daytime sleepiness, and increased perspiration [3]. Symptoms and signs in patients with acromegaly are slowly progressive and chronic, and these characteristics often lead to a delay in diagnosis, when the comorbidities are already irreversible or only partially reversible. The average delay in diagnosis ranges from 5 to more than 10 years after disease onset—although in most recent studies, this interval appears to have decreased—a period during which patients experience a progressive worsening of symptoms [3]. Diagnostic delay is due to the insidious nature of the disease with possible mild symptoms and signs that neither patients nor physicians attribute to a specific illness. The delay can also be related to an apparently normal GH concentrations or IGF-1 levels due to pre-existing illness, such as renal failure and diabetes mellitus, leading to false negatives upon testing [25]. Prolonged diagnostic delays are associated with a greater number of debilitating comorbidities and reduced QoL in patients [2,6,8,9,10,12]. Disease progression also depends on several factors such as histological classification, patients’ age (often younger patients have a more aggressive evolution than older but older patients may have the worst comorbidities), years of disease activity, adenoma features, and disease response to specific treatments [3].

The suspicion of acromegaly in a subject is based on the clinical manifestations and it can be confirmed with testing. The standard blood tests include IGF-1 levels and GH nadir after an oral glucose tolerance test. Imaging of the pituitary gland and magnetic resonance or computerized tomography (if MR is not applicable) are also used to confirm diagnosis. According to the guidelines, screening for excess of GH and IGF-1 is important in patients with clinical features of acromegaly associated with multimorbidity (e.g., type 2 diabetes mellitus, carpal tunnel syndrome, debilitating arthritis, hypertension, and sleep apnea) who are unresponsive to treatment. Similarly, if comorbidities that cause GH excess have an earlier than usual onset or if these comorbidities are associated with a pituitary mass (Katnelson ENDO guidelines, 2014), patients should also be tested for acromegaly.

Treatment of GH pituitary adenoma includes different therapeutic approaches, such as neurosurgery (the first-line treatment), medical treatments (first-generation somatostatin analogues, dopamine agonist, second-generation somatostatin analogue, GH receptor antagonist), and radiotherapy in select patients. Therapeutic goals in patients with acromegaly are the biochemical control of GH and IGF-1 secretion, the control of systemic comorbidities, the reduction of mortality, and the restoration of QoL.

## 4. Oral Manifestations and Maxillo-Facial Features

**Figure 1:**
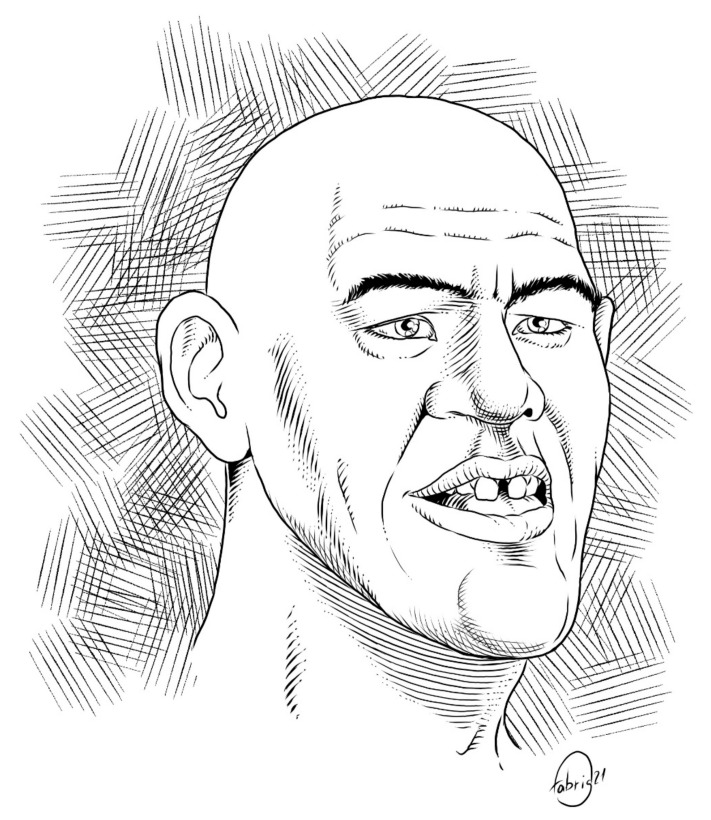
Oro-maxillo-facial features of acromegaly.

Among acromegaly symptoms, oral manifestations and maxillo-facial features are reported by most patients to have been present up to 10 years before diagnosis, preceded only by increased size of hands and feet. These manifestations may also be present in the later phases of the disease depending on duration of active disease, entity of diagnostic delay, and GH and IGF-1 secretion levels. Figure 1 and the following paragraphs provide a summary of the main facial dimorphisms described in the literature, including those that often lead to an acromegaly diagnosis [19].

**Figure 2:**
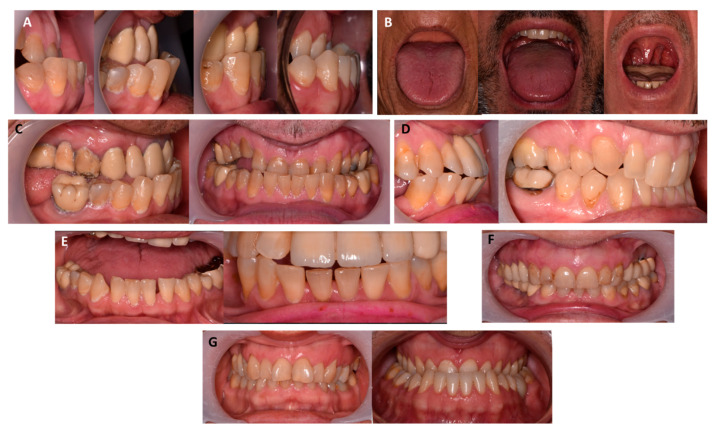
Oro-maxillo-facial features of acromegaly. Panel (**A**) Mandibular growth, prognathism, and class III dental and skeletal pattern; panel (**B**) macroglossia, hypertrophic uvula, and elongation of the soft palate; panel (**C**) dental occlusion; panel (**D**) inclined frontal elements, over-erupted molar teeth; panel (**E**) diastemas; panel (**F**) thickening of the gingival tissues; panel (**G**) palatine and/or mandibular torus and vestibular exostoses.

Mandibular growth, reported in 22–24% of patients, can lead to prognathism in 20–22% of these patients and the development of a class III dental and skeletal pattern (Figure 2, panel A) [1,6,9,19,20,22]. This growth is mainly based on the periosteal bone apposition due to the reactivation of the condylar growth centers [19]. Most studies affirm that the jawbone does not undergo particular alterations [26,27,28,29,30]. This growth, occurring after adolescence, can lead to unpredictable changes in the patient’s occlusal pattern, which should have already been consolidated, and can therefore result in painful symptoms, affecting the temporomandibular joint and masticatory muscles. The patient’s aesthetics can also be considerably altered by this late growth and can negatively impact the patient’s mental health and social relationships [19,20,22].

Macroglossia is seen in 54–58% of acromegaly patients [2,6,19,22]; further, the uvula may also be hypertrophic [25] and the soft palate may be elongated (Figure 2, panel B) [26]. All these alterations in the upper airways can result in sleep apnea, with a considerable negative impact on the quality of sleep and life of the patient [6,19,22]. Sleep apnea is commonly associated with multiple comorbidities related to the cardio-circulatory, endocrine, and nervous systems and has been associated with increased mortality [6,19,22]. Macroglossia and the hypertrophy soft tissue show the tendency of a spontaneous regression after surgical or medical treatment.

Acromegaly patients can also develop a dental malocclusion (Figure 2, panel C), which can lead to problems with dental prostheses, such as breaks or inconsistencies [20]. The loss of congruence of the patient’s dental prostheses can lead to fracture and incompatibility with the bone bases and may therefore require the restoration of the prostheses at a considerable financial cost to the patient [20].

Regarding dental elements, acromegaly patients may report diastemata (Figure 2, panel E), which occurs due to increased mandibular size and vestibular inclination of the teeth. This feature is reported in 40–43% of patients [1,6,9,19]. The presence of diastemas has been considered a pathognomonic factor of the disease and has even been included in ACROSCORE, a tool developed for diagnosing acromegaly [21]. The frontal elements may be inclined on the buccal or labial side due to macroglossia; molar teeth may be over-erupted, compensating for mandibular growth [24]. Some authors also believe that the dental elements can be characterized by a taurodontic aspect [19,27].

Some studies also describe an increased mineralization of the alveolar bone at the level of the roots of the molar elements, which outlines the features of hypercementosis [22,23,24,28,29].

The effects of the disease on the alteration of gingival tissues and on the development of periodontal and periradicular pathologies are still being debated, and the link with the disease remains questionable [22]. Some authors have reported the presence of a thickening of the gingival tissues in acromegaly patients (Figure 2, panel F) [22].

At the palatine and mandibular level, a study [22] identified the presence of torus and vestibular exostoses in a significant number of acromegalic patients, especially among younger subjects. If a patient needs prosthetics, this manifestation can lead to the necessity of oral surgery to remove the exostoses that would prevent the correct positioning and correct function of the prosthesis [22].

Clinically, not only dental components are modified by GH and IGF-1 excess but also soft tissue may be involved in facial changes due to acromegaly. In fact, it has been found that acromegalic patients commonly have enlarged submandibular glands (regardless of the activity of the disease) [31,32,33], though the parotid is rarely affected [34]. Glandular function is generally not compromised in these cases, and the pathophysiology related to these manifestations is not clear [34,35].

Finally, hypertrophy of the paranasal sinuses, especially of the frontal ones, may also occur. This aspect, together with laryngeal hypertrophy, can cause a deepening and sound resonance of the patient’s voice [1,6,9].

These oro-facial alterations in acromegaly patients may be associated with pain in the mouth, especially in the maxillo-facial area [21] and joint, which may be associated with joint clicks [20,22,25].

Biochemical treatment for the limitation of GH hypersecretion can only partially improve facial appearance, especially regarding soft tissues, but in most cases, oro-facial changes are not completely reversible and persist despite pharmacological treatment [19,25]. Table 1 provides a summary of the main oro-maxillo-facial features of acromegaly associated with the possible non-endocrinological treatments [36,37,38,39,40,41,42].

**Figure 3:**
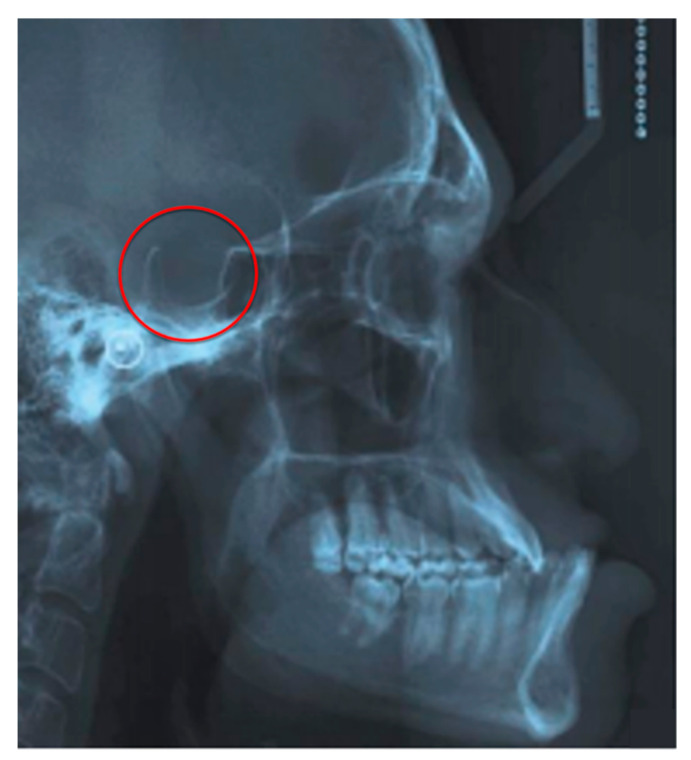
Cephalometric characteristics of a patient affected by acromegaly. Note the significant enlargement of sella turcica.

Some studies were also conducted using teleradiography examination to capture the cephalometric characteristics of acromegalic patients. The results showed that these patients generally report an increase in overall facial height and in the length of the cranial bases, an increase in the gonial angle, and an increase in the size of the branch [26] and of the mandibular body [26,43]. Overall, the mandible may be altered in shape [26,43,44,45,46]. The atlas cervical vertebra was found to be larger in these patients [43]. In some cases, the size of the sella turcica [45] may be evidently increased due to the dimensional alterations of the pituitary gland and the frontal sinuses may appear pneumatized (Figure 3). The frontal bones may also be thickened and the glabella may be protruding [26,43,44]. The size of the upper airways was substantially smaller in acromegalic patients, while the width of the soft palate was significantly greater, and the hyoid bone was positioned more vertically. Overall, teleradiography examination highlights the decrease in the pharyngeal space [43].

According to the literature, orthopantomography does not play a significant role in identifying the pathognomonic signs of the disease. It has been reported that only the panoramic and intraoral radiographs of these patients can reliably show radiopaque lesions at the level of the roots of the molar elements, defined as “hypercementosis” [23,28,29,43,47]. An observational study comparing the teleradiographs of the skulls of healthy subjects and acromegaly patients found that in a significant number of cases, the orthopantomography examination revealed morphological alterations of the diameter of the mandibular canal and enlargement of the mental foramen [48]. However, knowing that the orthopantomography and the teleradiographs are two-dimensional examinations, and often present distortions, this study cannot serve as a guide for the clinical diagnosis of the disease [49,50,51,52].

Nevertheless, clinicians should pay attention to these aspects when reading the results of radiographic examinations. Dentists and orthodontists [53] are advised not to focus their attention only on the teeth but to carefully examine every aspect of the oral cavity of each patient. On the other hand, these radiological examinations may be crucial also to non-dentist professionals to rule out or confirm the possibility of a pathological involvement of the pituitary sellar area.

## 5. Conclusions

About 80% of acromegaly patients present with oro-dento-facial signs, such as dental diastema, mandibular overgrowth, mandibular prognathism, and macroglossia. Dentists, orthodontists, and dental hygienists are in an ideal position to detect these signs early in acromegaly patients, which can lead to earlier diagnosis and improved prognosis. Moreover, with early diagnosis and subsequent appropriate endocrinological treatment earlier in the disease course, these signs can be significantly reduced. This would significantly impact patients’ QoL and self-esteem. It should be noted that even if the signs are not reduced by endocrinological treatment, they can be addressed with specific oral and maxillo-facial treatments.

For these reasons, oro-facial alterations, as distinctive and early signs of acromegaly, should be considered a primary target to be identified and evaluated properly. It is imperative to increase awareness among specialists, including dentists, orthodontists, and dental hygienists, with the specific goal of recognition of the pathology and earlier diagnosis of the disease. This would result in earlier initiation of treatment, which has been shown to improve patient outcomes in a myriad ways.

## Figures and Tables

**Table 1:** Oro-maxillo-facial features of acromegaly and possible treatments.

Symptoms/Signs	Diagnosis	Possible Treatments
Third-class malocclusion	Dental evaluation	No treatment or orthodontic treatment—if necessary, orthodontic-surgical treatment—in the inactive phase of the disease
Dental diastema	Dental evaluation	Possible conservative treatment in any phase of the disease, or orthodontic treatment, preferably not in the active phase of the disease
Macroglossia	Mallampati or Modified-Mallampati evaluation	Medical therapy for the control of acromegalic syndrome; possible surgical or conservative treatment of OSAS with special dental appliances
Osseous tori or exostoses	Dental evaluation	No treatment; surgical treatment in the event that the exostoses hinder the insertion of dental prostheses
Incongruity of mobile prostheses or implant-based prostheses, and possible breakage	Dental evaluation	Adjustment of the prosthetic bases or replacement of the same; in case of prostheses fixed on implants, verification and possible adjustment of the intraosseous pillars, preferably not in the active phase of the disease
Frontal elements inclination	Orthodontic evaluation	No treatment or orthodontic treatment in the inactive phase of the disease
Hypercementosis	Fortuitous, following routine radiographic examinations	No treatment
Clicks and TMJ pains	Dental and gnatologic evaluation, radiographic deepening with RM	Functional treatment with gymnastics for the temporomandibular joint or application of a customized bite The BruxApp app can be adopted under the control of the dentist to monitor and correct incorrect or flawed positions maintained during the day by the patient and to guide postural re-education
Thickening of gingival tissues	Dental evaluation	No treatment or surgical treatment in the inactive phase of the disease

## Data Availability

Not applicable.
